# Monitoring adherence to guidelines of antibiotic use in pediatric pneumonia: the MAREA study

**DOI:** 10.1186/s13052-017-0432-2

**Published:** 2017-12-22

**Authors:** Pasquale Di Pietro, Ornella Della Casa Alberighi, Michela Silvestri, Maria Angela Tosca, Anna Ruocco, Giorgio Conforti, Giovanni A. Rossi, Elio Castagnola, Maria Caterina Merlano, Simona Zappettini, Salvatore Renna, the Pediatric Ligurian Network MAREA network, R. Cambiaso, S. Cozzani, A. Defilippi, M. C. Diana, A. Ferrando, M. Fiore, D. Girosi, A. Insolvibile, G. Lepre, C. Muià, G. Picollo, G. Semprini, G. Squazzini, L. Tegaldo, A. Traverso, B. Tubino, G. Villa

**Affiliations:** 1 Pediatric Emergency Department, Istituto G, Gaslini, Genoa, Italy; 2 Clinical Pharmacology & Clinical Trials Unit, Istituto G, Gaslini, Genoa, Italy; 3 Pediatric Pulmonology and Allergy Unit and Cystic Fibrosis Center - Istituto G, Gaslini, Genoa, Italy; 4 Primary Care Pediatrician - FIMP Genoa, Genoa, Italy; 5 Infectious Disease Unit, Istituto G, Gaslini, Genoa, Italy; 6 Health Regional Agency of the Liguria Region, Genoa, Italy

**Keywords:** Antibiotic therapy, Pneumonia, Children, Appropriateness

## Abstract

**Background:**

Children are the most vulnerable population exposed to the use of antibiotics often incorrectly prescribed for the treatment of infections really due to viruses rather than to bacteria. We designed the MAREA study which consisted of two different studies: i) a surveillance study to monitor the safety/efficacy of the antibiotics for the treatment of pneumonia (CAP), pharyngotonsillitis and acute otitis media in children younger than 14 yrs old, living in Liguria, North-West Italy and ii) a pre−/post-interventional study to evaluate the appropriateness of antibiotic prescription for the treatment these infections. In this paper, we show only results of the appropriateness study about the antibiotic prescription for the treatment of pneumonia.

**Methods:**

Patients included in this study met the following inclusion criteria: i) admission to the Emergency/Inpatient Dpt/outpatient clinic of primary care pediatricians for pneumonia requiring antibiotics, ii) informed written consent. The practice of prescribing antibiotics was evaluated before-and-after a 1 day-educational intervention on International/National recommendations.

**Results:**

Global adherence to guidelines was fulfilled in 45%: main reason for discordance was duration (shorter than recommended). Macrolide monotherapy and cephalosporins were highly prescribed; ampicillin/amoxicillin use was limited. 61% of patients received >1 antibiotic; parenteral route was used in 33%. After intervention, i) in all CAP, cephalosporin prescription decreased (−23%) and the inappropriate macrolide prescriptions was halved and, ii) in not hospitalized CAP (notH-CAP), macrolides were prescribed less frequently (−25%) and global adherence to guidelines improved (+39%); and iii) in H-CAP antibiotic choice appropriateness increase.

**Conclusion:**

Prescribing practices were sufficiently appropriate but widespread preference for multidrug empirical regimens or macrolide in monotherapy deserve closer investigation.

## Background

Drug prescribing attitude varies from country to country probably related to the needs of different patient populations and to the individual attitudes of decision making among physicians [1]. Antibiotics are one of the most prescribed drugs throughout the world and their use appears to be significantly conditioned by patients’ demand and by other social factors [1, 2]. In Western countries, the most frequent indications for the prescription of antibiotics are upper and lower respiratory tract infections such as pneumonia both in adults and in children [3]. Unguided use can lead to drug wastage, lost in confidence in efficacious antibiotics, superinfection, ineffective treatment, delayed healing, unnecessary dependency from medical aid, spread of resistant organisms [4–6]. Among developed countries, Italy has one of the highest rates of antibiotic use [7]. Despite availability of guidelines also for the treatment of pediatric pneumonia [5, 8], overprescription/abuse of antibiotics in the treatment of respiratory infections is still a worldwide problem [9, 10]*.* Also the Regional Health Plan of Liguria, promotes the reduction of the use of antibiotics and especially of last generation antibiotics when appropriate therapies equally effective against the etiologic agents are available. Educational interventions seem to be effective in improving a more rationale use of antibiotics [11–14]. Children are the most vulnerable population exposed to the use of antibiotics often incorrectly prescribed for the treatment of infections such as pneumonia, we designed the MAREA study (far**MA**covigilanza in pediatria **RE**gione Liguri**A** i.e. pharmacolovigilance in pediatric patients in Ligurian Region): one of the aim of this study was to evaluate the appropriateness of antibiotic prescription for the treatment of pneumonia before and after an educational intervention. The findings of this study may further confirm that priority actions for improving the rational use of antibacterials in children should concentrate on education activities such as active participation of the pediatricians to training courses.

## Methods

### Study design

The MAREA study comprised two different studies with different designs: i) a phase IV, time-cohort, multicenter 2 yr-surveillance study to monitor the safety/efficacy of the antibiotics for the treatment of pneumonia, pharyngotonsillitis and AOM in Liguria (Italy) and ii) a pre−/post-interventional study to evaluate the appropriateness of antibiotic prescription for the treatment these infections.

In this paper, we show only results of the appropriateness study about the antibiotic prescription for the treatment of pneumonia.

Briefly, for the surveillance study, patients were followed-up for 21 days with the aim to evaluate the frequency of i) hospitalization for pneumonia, pharyngotonsillitis or AOM, ii) the occurrence of adverse drug reactions (ADR), iii) the hospitalization for ADR/serious adverse events.

The appropriateness study was designed to collect data on the disease management and antibiotic prescriptions and to evaluate possible effect of an educational intervention to increase appropriate management and antibiotic prescriptions.

A web-based electronic case report form (e-CRF) was used to collect all data anonymously. Briefly, after having obtained written informed consent by parents/tutors, each investigator uploaded data in a web-based e-CRF. To access the e-CRF, each investigator was requested to enter her/his login/password. Private data (such as first and last name or date of birth) were automatically encrypted before sending to the central server (so data were anonymously collected).

If during the follow-up, a patient needed to be visited by a physician different from the “enrolling” physician, data entered at the enrollment was accessible also to the “follow-up” physician who could update the e-CRF with additional information. Demographic (age and gender), antropometric (i.e. weight) and clinical data (i.e. diagnostic procedure and details of antibiotic administration) were recovered.

The surveillance and the appropriateness studies were approved by Ethics Committee of the Gaslini Institute. For the surveillance study, all participants and their parents or tutors were informed in detail on the experimental procedure and the main scope of the study and provided written informed consent: For the appropriateness study, in the pre-intervention phase, pediatricians were kept unaware of the aim of the study.

### MAREA patient population and setting

Seventeen pediatric physicians (9 hospital physicians and 8 primary care pediatricians) belonging to the MAREA Network participated in the study and enrolled patients with the following characteristics: i) aged <14 yrs old, male and female; ii) patients admitted to the Emergency/Inpatient Dpt or in outpatient clinic of family physicians with a diagnosis of pneumonia, pharyngotonsillitis or AOM requiring antibiotic treatment and iii) informed written consent. Exclusion criteria were as follows: cystic fibrosis, cancer, immune deficiencies, living outside Liguria.

Patients were enrolled during two periods (each of 11 months from February to December) (phase I: 2013 and phase II: 2014): between the two phases a 1-day educational intervention on the diagnosis and antibiotic treatment of respiratory tract infections was performed.

In this paper, we show only data on patients enrolled with a diagnosis of pneumonia.

### Training between phase I and II of the study

This intervention consisted of 1-day educational sessions on the diagnosis and treatment of each of the three indications according to guidelines (Table 1). Briefly, all 17 physicians were exposed to the education intervention (all in one place). Some members of the MAREA Steering Committee (2 pediatric pulmonologists, 1 pediatric infectious disease specialist, 2 pediatric and medical emergency specialist and, 2 primary care pediatricians) provided educational sessions for the diagnosis and the treatment of pneumonia with specific recommendations for the best prescribing practices (International/National recommendations). On this occasion, the results of the first phase of the study were reported. Of note that in the first phase of this study, pediatricians were kept unaware of the evaluation, so their prescribing of antibiotic practices could not have been influenced by the study. Each session was followed by a 15–30 min discussion of the guideline indications and of the results obtained. In addition, each physician received a copy of the slides that were shown and concise written materials with focused guidance on pneumonia. The second phase took place immediately after the educational intervention. Monthly update about the enrolment rate in the second phase was sent to the physician.

**Table 1:** Drug treatments for pneumonia in children as suggested by Esposito S et al. (Esposito et al., 2012), revised

Antibiotic choice	Dose and route of administration	Total duration of treatment
*First choice*		
Amoxicillin	50–90 mg/kg/d (max 3.000 mg) in 2–3 doses. Oral	5–7 days
Ampicillin	100 mg/kg/d (max 12.000 mg) in 3–4 doses. iv	5–7 days
Clarithromycin	15 mg/kg/d (max 1.000 mg) in 2 divided doses. Oral	14 days
Clarithromycin	4–8 mg/kg/day in 2 divided doses. iv	14 days
Azithromycin	10 mg/kg/d (max 500 mg) in 1 dose. Oral	3 days
	1 dose of 10 mg/kg/d and then 5 mg/kg/d. oral	4 days
*Second choice*		
Amoxicillin/clavulanate	amoxicillin component: 50–90 mg/kg/d (max 3.000 mg) in 2 doses. Oral	5–7 days
Ampicillin/sulbactam	150 mg/kg/d (max 9.000 mg) in 3 doses. iv	
Cefuroxime axetil	20–30 mg/kg/d in 2 divided doses. Oral	
Benzylpenicillin iv	200.000 units/kg/d in 4–6 doses. iv	
Ceftriaxone	80–100 mg/kg (max 2.000 mg) once a day. iv	
Cefotaxime	100–150 mg/kg/d in 3 divided doses. iv	
Cefaclor	20–40 mg/kg once a day. Oral	
Cefpodoxime proxetil	8 mg/kg once a day. Oral	

Combination therapy with a beta-lactam drug and a macrolide can be considered in more severe cases or when an atypical bacterial infection is suspected

### Definition of pneumonia

According to the clinical findings, children were diagnosed as pneumonia if they had recurrent or persistent fever (>38.5 °C) associated to chest indrawing and increased respiratory rate [5]. Pneumonia was confirmed by chest X-rays. (that were performed in almost all patients hospitalized children i.e. in 135 out of 137).

### Criteria for admission

The appropriateness of the disease management i.e. for example the decision of hospitalizing patients, will be reported in a future paper. Briefly, the following clinical and demographic characteristics were collected: i) respiratory difficulty, ii) local hypophonesis/localized crackles, iii) recurrent/persistent body temperature ≥ 38.5 °C, iv), dehydration, v) thoracic and/or abdominal pain, vi) O_2_ saturation < 92% (in children aged <5 yrs. old, <95%) vii) tachidyspnoea, viii) persistent dry cough, ix) nasal flaring, x) cyanosis, xi) comorbidities, xii) age ≤ 1 yr. old, xiii) inability to feed her/himself, xiv) poor familiar compliance.

### Antibiotic prescription

The practice of prescribing antibiotics was evaluate before and after a 1 day-educational intervention on the diagnosis and antibiotic treatment of respiratory tract infections in children based on International/National recommendations [5, 8] (Table 1)

The appropriateness of the antibiotic prescriptions was assessed by comparing the clinical practice of each physician with consensus guidelines developed for pneumonia and presented at the educational sessions. The following aspects were assessed: i) indication—appropriate decision making regarding use/nonuse of antibiotics; ii) antibiotic choice; iii) total duration of antibiotic use; iv) dose. We considered as appropriate a choice of an antibiotic independently if it was a “first” or a “second” choice.

The global adherence of physicians’ antibiotics prescription was considered appropriate when totally according to the current clinical practice guidelines and any divergence from the guidelines led to a final assessment of the treatment course as discordant with the guidelines.

Each parameters was also evaluated separately so that the nonadherence to one parameter or missing data for one parameter did not preclude assessment of the others. If more than 1 drug was prescribed, total duration of antibiotic use and dose were evaluated separately for each drug.

Appropriateness of prescriptions was expressed as percentage.

### Statistical analysis

The needed sample size was calculated, assuming an expected frequency of hospitalization of about 10% in pediatric population with a diagnosis of pneumonia, pharyngotonsillitis and/or AOM, the type I error = 0.05, the power = 0.80 and the exact binomial test. With these assumptions, the needed sample size ranges was 840 patients (420 patients each phase). Being the appropriateness study strictly depend on the surveillance study (i.e. the number antibiotics prescriptions are related to the number of patients enrolled), the same sample size was kept also for the appropriateness study. The needed sample size and power was calculated according to Gatsonis C and Sampson AR formulations and using the Software NQuery Advisor (release 7.0).

Descriptive statistics of the characteristics of the patients were performed and reported in terms of mean and standard deviation (SD) for the quantitative variable i.e. chronological age and in terms of absolute frequencies and percentages for the qualitative variables (i.e. antibiotic prescriptions).

Comparison of frequency data was performed by the chi-square test or by the Fisher’s exact test in case of expected frequencies less than five. All tests were two-sided and a *P* value less than 0.05 was considered statistically significant. “Statistica release 6” (StatSoft Corp., Tulsa, OK, USA) was used.

## Results

Analyzing the whole MAREA population (i.e. those with pneumonia, pharyngotonsillitis and/or AOM), we found a higher frequency of hospitalization for the three indications (i.e pneumonia, pharyngotonsillitis or AOM) than expected (25% vs. 10%).

Demographic characteristics of the study population (*N* = 225) are reported in Table 2.

**Table 2:** Demographic and clinical characteristics of the study population

	All (No.225)	Hospitalized pneumonia (No.137)	Not-hospitalized pneumonia (No.88)
Age [months (mean (SD)]	60.73 (34.03)	55.99 (32.00)	68.06 (35.93)
Gender (male-to-female ratio)	1.06	0.89	1.38
Enrollment in phase I [No. (%)]	142 (63.11)	94/142 (66.20)	48/142 (33.80)
		94/137 (68.61)	48/88 (54.55)
Enrollment in phase II [No. (%)]	83 (36.89)	43/83 (51.81)	40/83 (48.19)
		43/137 (31.38)	40/88 (45.45)

Overall, a high number of patients with pneumonia were severe enough to require hospital admission (No. 137, 60.9%): 66.2% phase I and 51.8% in phase II.

### Antibiotic prescription before-and-after educational intervention in the whole population of children with pneumonia and in hospitalized (H-CAP) and not-hospitalized pneumonia (NotH-CAP)

**Fig. 1:**
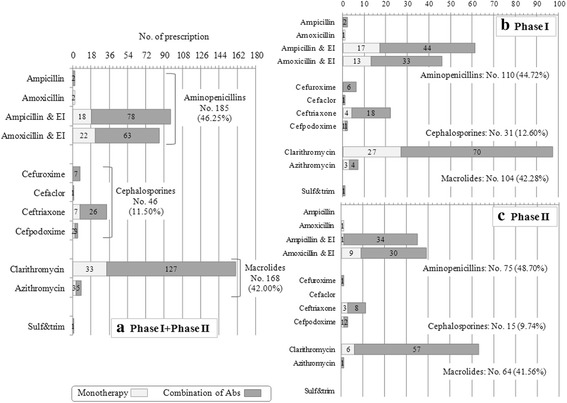
Antibiotic prescription for the treatment of pneumonia in all the studied period (panel **a**) or before (phase I, panel **b**) and after 1 day-educational intervention (phase II, panel **c**). Type of antibiotics is reported on the ordinate and the number of prescription on the abscissa

In all, 225 children enrolled for pneumonia received a total of 400 antibiotic prescriptions. Distribution of prescriptions by antibiotic therapeutic category (aminopenicillins, cephalosporines and macrolides) and by antibiotic molecule are reported in Fig. 1. Aminopenicillins and macrolides were the most frequently used class of antibiotics (Fig. 1a) both in phase I (Fig. 1b) and in phase II (Fig. 1c). Cephalosporins were prescribed in 11.5% (46 prescriptions) of the patients (Fig. 1a), 23% lower in phase II (phase I: 31 prescriptions, 12.6%, phase II: 15 prescriptions, 9.7%, *p* = .38) (Fig. 1b and c).

Clarithromycin (160 prescriptions, 40.0%) followed by ampicillin + enzyme inhibitor (EI) (96 prescriptions, 24.0%) or amoxicillin + enzyme inhibitor (EI) (85 prescriptions, 21.3%) were the most frequently prescribed drugs (Fig. 1a). Ampicillin/amoxicillin (without EI) were prescribed only in 4 cases (Fig. 1a).

About 61% of children (No. 138) received more than one antibiotic: the most frequent prescriptions was macrolide and aminopenicillins + EI (No. 111/138, 80.4%) both in phase I (No. 60/76, 79.0%) and in phase II (No. 51/62, 82.3%). About 50% of children who received more than one antibiotic was treated with a combination of macrolide and ampicillin + EI (No. 73/138, 52.9%): this combination was prescribed about 7-fold more frequently in H-CAP children (No. 69/99, 69.70%) than in notH-CAP children (No. 4/39, 10.26%) (*p* < .0001). In addition, macrolide was more frequently prescribed with ampicillin + EI in H-CAP (69/99 prescriptions, 69.70%) and to amoxicillin + EI in notH- CAP (34/39 prescriptions, 87.18%).

Macrolide monotherapy was prescribed in 36 cases (9% of total prescriptions).

**Fig. 2:**
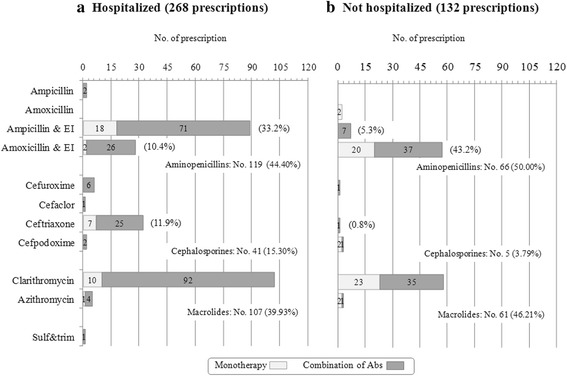
Antibiotic prescription for the treatment of pneumonia requiring hospitalization (panel **a**) or not requiring hospitalization (panel **b**). Type of antibiotic is reported on the ordinate and the number of prescription on the abscissa

Comparing H-CAP and notH-CAP, we found a statistically significant difference in the distribution of antibiotic therapeutic category prescriptions (*p* = .003): cephalosporins were prescribed 5-fold more frequently in H-CAP (Fig. 2a) than in notH-CAP (Fig. 2b) being 15.30% (41/268 prescriptions) vs 3.79% (5/132 prescriptions), respectively: H-CAP patients were more likely to receive cephalosporins as compared to notH-CAP (*OR*: 4.59 [95%CI: 1.77–11.90], *p* = .0007). Differently, the frequency of prescriptions of aminopenicillins and macrolides were similar in the two groups (Fig. 2). Analyzing the distribution of prescriptions by antibiotic molecule, we found that ampicillin + EI (*p* < .001) and ceftriaxone (*p* = .0001) were administered significantly more frequently in H-CAP (Fig. 2a) whereas amoxicillin + EI (*p* < .001) more frequently in notH-CAP (Fig. 2b).

A combination of antibiotics was used more frequently to treat H-CAP patients (72.26%, 99/137) than notH-CAP children (44.32%, 39/88) (*p* = .0007).

**Fig. 3:**
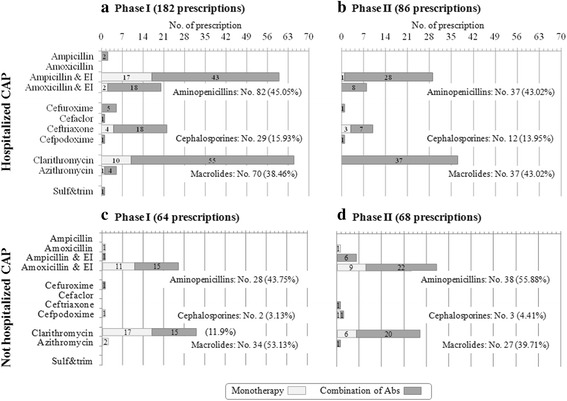
Antibiotic prescription for the treatment of pneumonia requiring hospitalization (panels **a** and **b**) or not requiring hospitalization (panels **c** and **d**) before (panels **a** and **b**) and after (panels **b** and **d**) 1 day-educational intervention. Type of antibiotics is reported on the ordinate and the number of prescription on the abscissa

In H-CAP, no difference in the prescription of each class of antibiotics was seen between phase I and phase II, (Fig. 3a and b). In notH-CAP, educational intervention was followed by a 25% decrease in macrolide prescription (phase I: 34/64 prescriptions, 53.13% vs. phase II: 27/68 prescriptions, 39.71%, *p* = .12) (Fig. 3c and d).

About two-third of prescriptions were for oral use (268/400 prescriptions), both in phase I and in phase II (Table 3), especially macrolides (268/400 prescriptions, 62.31%) followed by ampicillin/amoxicillin + EI (87/400 prescriptions, 32.46%).

**Table 3:** Route of administration for antibiotics in hospitalized (H-CAP) and not-hospitalized (notH-CAP) patients before and after educational course

	Route of administration for antibiotics		*P* value	
	Oral [No (%)]	Parenteral [No (%)]	Before vs. after	H-CAP vs. notH-CAP
*Prescriptions for the whole study population (No. 400)*	268 (67.00)	132 (33.00%)		
Before educational course *(No. 246)*	160 (65.04)	86 (34.96)	0.29	
After educational course *(No. 154)*	108 (70.13)	46 (29.87)		
*Prescriptions for hospitalized patients* (No. 268)	144 (53.73)	124 (46.27)		< .0001
Before educational course *(No. 182)*	97 (53.30)	85 (46.70)	0.84	< .001
After educational course *(No. 86)*	47 (54.65)	39 (45.35)		< .001
*Prescriptions for not-hospitalized patients* (No. 132)	124 (93.94)	8 (6.06)		
Before educational course *(No. 64)*	63 (98.44)	1 (1.56)	0.06	
After educational course *(No.68)*	61 (89.71)	7 (10.29)		

The parenteral route accounted for 33.00% of prescriptions (Table 3), and it was more commonly use to administer antibiotics to H-CAP (46.27%) than to notH-CAP (6.06%) children (*p* < .0001) (Table 3); among parenteral antibiotic, ampicillin alone or added to sulbactam was the most frequently administered (98 prescriptions, 74.24% of all parenteral prescriptions), followed by third-generation cephalosporins (33 prescriptions, 25.00%).

Patients were treated by parenteral antibiotics for a mean of 6.28 (SD: 3.77) days, a longer time of treatment if they were hospitalized for pneumonia [6.66 (SD:3.66) days] than if they had milder pneumonia [1.75 (SD: 1.49) days] (*p* = .001). In phase II, a slight decrease in parenteral antibiotic prescription was observed but without reaching statistically significance (34.96% vs. 29.87%, respectively, *p* = .29).

#### Adherence to guidelines: Assessment of individual parameters

Parameters were evaluated separately, so that nonadherence to one parameter did not preclude assessment of the others.

#### Indication and antibiotic choice

The decision making regarding use or nonuse of antibiotics (i.e. indication) was appropriate in all cases.

Antibiotic choice was adherent to the recommendations in 92.50% of the cases without statistically significant difference between phase I and phase II (*p* = .17) (Table 4). Adherence to guidelines was higher for H-CAP than for not-H-CAP (96.27% vs. 84.85%; *p* < .001) (Table 5); this was true both in phase 1 (H-CAP: 172/182 prescriptions, 94.51% vs. not-H-CAP: 52/64 prescriptions, 81.25%; *p* = .001) and in phase 2 (H-CAP: 86/86 prescriptions, 100% vs. not-H-CAP: 60/68 prescriptions, 88.24%; *p* = .001). In addition, in H-CAP, we found a significant increase after the educational intervention and the adherence to guidelines reached 100% (*p* = .03) (Table 6). Differently, in notH-CAP, appropriateness rate for antibiotic choice remained almost stable in phase II (phase I: 81.25%, phase II: 88.24%, *p* = .26) (Table 6).

**Table 4:** Frequency of appropriateness of antibiotic use in children with pneumonia evaluated before and after educational course

	Children with pneumonia (phase I + II) (No. 400 prescriptions)	Pre-educational course (phase I) (No. 246 prescriptions)	Post-educational course (phase II) (No. 154 prescriptions)	*P* value
Single parameter appropriateness				
*Indication* [No. (%)]	400 (100.00)	–	–	–
*Choice of antibiotic* [No. (%)]	370 (92.50)	224 (91.06)	146 (94.81)	.17
*Duration* [No. (%)]	242/389^a^ (62.21)	143/236^a^ (60.59)	99/153^a^ (64.71)	.41
*Dose* [No. (%)]	324/399^a^ (81.20)	198/245^a^ (80.82)	126 (81.82)	.80
Global appropriateness [No. (%)]	175/389^a^ (44.99)	103/236 (43.64)	72/153 (47.06)	.51

^a^when data are lacking, the total number of data available are reported

**Table 5:** Frequency of appropriateness of antibiotic use in children with pneumonia requiring or not requiring hospitalization

	Children with pneumonia (No. 400 prescriptions)	Hospitalized CAP (No. 268 prescriptions)	Not hospitalized CAP (No. 132 prescriptions)	*P* value
Single parameter appropriateness				
*Indication* [No. (%)]	400 (100.00)	–	–	–
*Choice of antibiotic* [No. (%)]	370 (92.50)	258 (96.27)	112 (84.85)	< .001
*Duration* [No. (%)]	242/389^a^ (62.21)	162/261^a^ (62.07)	80/128^a^ (62.50)	.93
*Dose* [No. (%)]	324/399^a^ (81.20)	235/267^a^ (88.01)	89 (67.42)	< .001
Global appropriateness [No. (%)]	175/389^a^ (44.99)	132/261 (50.57)	43/128 (33.59)	.0016

^a^when data are lacking, the total number of data available are reported

**Table 6:** Frequency of appropriateness of antibiotic use in children with pneumonia (CAP) requiring hospitalization or not requiring hospitalization evaluated before (phase I) and after (phase II) educational course

	Hospitalized CAP (H-CAP)				Not hospitalized CAP (NotH-CAP)				*P* value
	Phase I + II (No. 268 prescriptions)	Phase I (No. 182 prescriptions)	Phase II (No. 86 prescriptions)	*P* value (phase I vs II)	Phase I + II (No. 132 prescriptions)	Phase I (No. 64 prescriptions	Phase II (No. 68 prescriptions)	*P* value (phase I vs. II)	H-CAP vs. notH-CAP
Indication	268 (100.00)	–	–	–	132 (100.00)	–	–	–	–
Choice of antibiotic	258 (96.27)	172 (94.51)	86 (100.00)	.033	112 (84.85)	52 (81.25)	60 (88.24)	.26	< .001^b,c^
Duration	162/261^a^ (62.07)	111/175^a^ (63.43)	51 (59.30)	.52	80/128^a^ (62.50)	32/61^a^ (52.46)	48/67^a^ (71.64)	.025	.93
Dose	235/267^a^ (88.01)	156/181^a^ (86.19)	79 (91.86)	.18	89 (67.42)	42 (65.63)	47 (69.12)	.67	< .001
Global appropriateness	132/261^a^ (50.57)	86/175 (49.14)	46 (53.49)	.51	43/128 (33.59)	17/61 (27.87)	26/67 (38.81)	.19	< .001

All data are reported as No. (%)

^a^when data are lacking, the total number of data available are reported

^b^comparison between phase I in H-CAP and notH-CAP

^c^comparison between phase II in H-CAP and notH-CAP

Comparing the frequency of inappropriateness in antibiotic choice for the different antibiotic classes, we found that in phase I the frequency of inappropriate prescriptions was higher for macrolides than for other antibiotics (i.e. aminopenicillins or cephalosporines) (14/104 prescriptions vs. 8/142 prescriptions, 13.46% vs 5.63%, respectively, *p* = .034). In phase II, the frequency of inappropriate prescriptions was further reduced and became similar being 7.81% (5/64 prescriptions) for macrolides and 3.33% (3/90 prescriptions) for other antibiotics (*p* = .22). This was mainly due to a 45% reduction in inappropriate macrolide prescription in phase II even though not statistically significant (*p* = .26), (data not shown).

#### Total duration of antibiotic use

In 62.21% of prescriptions, total duration of antibiotic use was concordant with the guidelines; educational intervention only slightly increase adherence (*p* = .41) (Table 4). Inappropriate duration was seen only for macrolides that were used shorter than recommended [mean duration (days) 9.65 (SD: 1.76); min:3, max: 13]: the mean duration of antimicrobial treatment with macrolides was 9.31 (SD 3.04) days in hospitalized and 9.83 (SD 2.49) days in not-hospitalized patients (*p* = 0.27). In notH-CAP, a 36% increase in guideline adherence of duration of treatment was observed in phase II (*p* = .025) (Table 6).

Analyzing only macrolide prescriptions appropriate for choice, we found that only 13.19% of them were concordant to the guidelines (19/144 prescriptions) also for duration with an about 30% increase in adherence in phase II but without reaching statistically significance (phase I: 10/85 prescriptions, 11.76%, phase II: 9/59 prescriptions, 15.25%, *p* = .54). In notH-CAP, a 5-fold increase in adherence also for duration was found after the educational intervention being 4.76% (1/21 prescriptions) before and 27.27% (6/22 prescriptions) after but this enhancement was not statistically significant (*p* = .095).

#### Dose

The dose of antibiotics was concordant with the guidelines in 81.20% (324 prescriptions) of the 399 evaluated doses (1 case missing) (Table 4). In the discordant doses (75 prescriptions), the dose per kilogram was lower than that recommended in 66.67%. Educational intervention did not modify the appropriateness rate for this parameter (Table 4) both in H-CAP (Table 6) and in notH-CAP (Table 6).

Concordant doses were found more frequently in H-CAP than in notH-CAP being 88.01% and 67.42%, respectively (*p* < .001) (Table 5); this was true both in phase I (*p* = .0003) and in phase II (*p* = .0003) (data not shown).

### Global adherence to guidelines

Global adherence to guidelines *for all parameters* was fulfilled in 45% of the cases. Age did not affect the frequency of concordant prescriptions (not shown). No statistically significant difference between phase I and phase II was found both in all CAP and in H-CAP (Tables 4 and 6). Differently, in notH-CAP, the educational intervention determined a 39.26% improvement in global adherence to guidelines without reaching statistically significance (phase I: 27.87%, phase II: 38.81%, *p* = .19) (Table 6).

Analyzing inappropriate prescriptions (No = 220), the main reason for discordance was duration of treatment (147 prescriptions, 66.81% of all 220 discordant cases), followed by wrong dose (75 prescriptions, 34.09% of discordant cases) and antibiotic choice (30 prescriptions, 13.64% of discordant cases). Similar results were obtained analyzing the two phases separately (not shown).

Since the most common deviation from the guidelines was duration, we re-evaluated “global appropriateness” only considering concordance/discordance for indication, antibiotic choice and dose. We found an almost 60% of adherence to guidelines (235 prescriptions, 58.75%). Again, in notH-CAP, after educational intervention, appropriateness significantly increased from 46.88% to 64.71% (30/64 prescriptions to 44/68 prescriptions, respectively; *p* = .039).

No adverse event was reported.

## Discussion

The main findings of this study are: i) the decision making regarding use/nonuse of antibiotics was appropriate in all cases but global adherence to guidelines was fulfilled in less than a half of the cases and the main reason for discordance was duration of treatment; ii) cephalosporins and macrolide monotherapy were highly prescribed whereas use of unprotected ampicillin/amoxicillin was very limited; iii) a high percentage of patients received more than one antibiotic and parenteral route was used in over one third of prescriptions; iv) *after educational intervention*: inappropriate macrolide prescriptions was halved; in notH-CAP, cephalosporin and macrolide prescriptions decreased, global adherence to guidelines and appropriateness of duration of treatment improved; in H-CAP, antibiotic choice appropriateness rate increased.

Despite availability of guidelines, overprescription/abuse of antibiotics in the treatment of respiratory infections is still a worldwide problem [9, 10]*.* About 50% of antibacterial prescriptions for children are inappropriate [15, 16] and, in low-income countries, this percentage is even higher [17].

There are several reasons for these unjustified prescriptions: i) to be conditioned by parental pressure to receive antibiotics [1, 2, 11, 18]; ii) the idea that antibiotic use can be more effective than diagnostic tests and observation of patient evolution [18] and may prevent bacterial superinfections and their complications [18–20]; iii) inertia related to previous practice patterns; iv) lack of awareness, inadequate knowledge, or perceived ineffectiveness of the guidelines [21].

In our studied population, the decision making regarding use/nonuse of antibiotics was appropriate in all cases. However, a rational drug use occurs not only when an appropriate drug is prescribed but also when it is administered in correct doses and for an adequate duration of therapy [22]. Several studies investigated not only the appropriateness of the choice of the molecule but also the duration of use and the dose, but all of them included adult population or patients with other diseases [23–25]. We evaluated a “global” adherence to guidelines of antibiotic use according to the document approved by the consensus conference for the treatment of respiratory tract infections in children which promoted not only the rational use of antibiotics, but also focusing on the appropriate agent, dosage and optimal duration of antibiotic therapy [8]. Before the educational training, adherence to guidelines in antibiotic use for all parameters was fulfilled in less than a half of the cases and the main reason for discordance was duration of treatment, that was shorter than recommended. Other healthcare settings in adult population reported similar results: adherence rates to guidelines of 44–49% have been reported in ambulatory patients with community-acquired pneumonia [26].

Assessing individual parameters, antibiotic choice was adherent to the recommendations in over 90% of the cases and was higher for H-CAP. In phase I, macrolides were inappropriately prescribed more frequently than other antibiotics (13.5% vs 5.6%). In a retrospective analysis of 1 year-ambulatory pediatric visits, Saleh et al. [27] found similar results in children with pneumonia: macrolides were not recommended in 22.5% of cases. Similarly, Kronman et al. [28] found that the use of macrolides ranges from 27.8% to 41.7% of all antibiotics prescribed for childhood CAP and this percentage increased 10.0% every 2 years over the entire 14 year-study. The frequent use of macrolides should be discouraged because it can increase the burden of antibiotic resistance among pneumococci [29, 30]. According to European surveillance data, the frequency of macrolide resistance among pneumococci isolates from Italy [29] is already the one of highest in Europe [31] being between 10% and 29%. In Italy, regardless the disease, cephalosporins are widely prescribed, while they represent less than 1% of the pediatric prescriptions in Denmark and the Netherlands [32]. Also our study confirms a relatively high cephalosporin use being prescribed in 11.5% of cases, predominantly in hospitalized patients (15% vs 3%). This result together to the relatively high rates of macrolide monotherapy (9%) and the very low rates of use of unprotected ampicillin (0.75% in H-CAP) or amoxicillin use (1.52% in notH-CAP) highlight a need for effective initiatives to improve the use of antimicrobial agents. Indeed, amoxicillin is considered the first-line antibiotic for the most common paediatric infectious diseases. In Italy, amoxicillin represents about one-fifth of antibiotic paediatric prescriptions, whereas in Canada and in the Netherlands, it represents half of the prescriptions [33]. In Italy, amoxicillin/clavulanate prescriptions are 3-fold higher than in the Netherlands and 5-fold higher than in Canada [33] and the trend is increasing. The preference of amoxicillin/clavulanate is mainly due to the fear of infections caused by beta-lactamase-producing bacteria.

Of note that more than 60% of patients received more than one antibiotic similarly to the results reported by DeLuca et al. [34] who found that 50% of LRTI cases received more than one antibiotic. The most frequent prescriptions was macrolide and aminopenicillins + EI. This may be explained considering that even though the main cause of pneumonia is *Streptococcus pneumonia* (the resistance of *Streptococcus pneumoniae* could be overcome by high dose of ampicillin (up to 80 mg/kg) rather than by adding of clavulanate), also pneumonia due to *Mycoplasma pneumoniae* is not very unusual in preschool children [35].

Despite the fact that there are no evidence supporting the superiority of intravenous/intramuscular treatment of pneumonia as compared to the oral one, in our study parenteral route accounted for about one third of prescriptions and among them ampicillin alone or added to sulbactam was the most frequently administered (74.2% of all parenteral prescriptions), followed by third-generation cephalosporins (25.0%). An abuse of parenteral cephalosporins in Italian hospitalized children was already denounced in a study conducted last decade by Esposito et al. [36]. Parenteral administration may be justified by the clinical conditions of the patients: oral absorption of drug may be disturbed due to vomiting or in severely ill patients.

We also reported that duration of use and dose of antibiotics was concordant with the guidelines in over 60% and in over 80%, respectively. Inappropriate duration was seen only for macrolides that were used shorter than recommended (mean: 9.6 days. 9.3 days in hospitalized and 9.8 days in not-hospitalized patients). - the guidelines we refer suggested to use macrolides for 14 days but other recommendations such as guidelines by the Pediatric Infectious Diseases Society and the Infectious Diseases Society of America suggests to prescribe a shorter course of antibiotic treatment at least in milder pneumonia [37]. In our sample population of not hospitalized CAP, the duration of treatment was longer [9.8 (SD 2.5) days] than that suggested by Pediatric Infectious Diseases Society and the Infectious Diseases Society of America guidelines. In discordant doses, the dose per kilogram was lower than that recommended in 67.6% of the cases. Concordant doses were found significantly more frequently in H-CAP than in notH-CAP being 88.01% and 67.42%, respectively.

On the basis of these results, guidelines alone seem to have a limited efficacy in changing physician behavior in the management of pediatric pneumonia, probably because primary care pediatricians have been faced with a variety of Italian/international guidelines, sometimes with contrasting key messages, requiring substantial time and effort to be analyzed and followed. Therefore, paediatricians knowledge and approach to antibiotic use require corrections to improve prescribing practices [11–14, 38]. Educational campaigns/interventions promoting the rational use of antibiotics have been demonstrated to be effective in improving physician and nurse practitioner behavior [11–14] and particularly, if providing concise written materials with focused guidance on specific diseases [11]. Neuman and colleagues [39] recently demonstrated that after an introduction of an antimicrobial stewardship program and a clinical practice guidelines, the use of ampicillin increased by 34%, whereas ceftriaxone use was halved. Similarly, it has been reported a greater use of narrow-spectrum antibiotics, such as penicillin and aminopenicillins, among children admitted to hospitals with clinical practice guidelines as compared to those without guideline [39]. After educational intervention, we found a 23% decrease in cephalosporin prescriptions in all CAP and a 25% decrease in macrolide prescription in notH-CAP.

Global adherence to guidelines was similar in the two phases of the study. However, analyzing antibiotic prescriptions in notH-CAP, the educational intervention determined an about 44% improvement in overall adherence to guidelines. Parameters were also evaluated separately, so that nonadherence to one parameter did not preclude assessment of the others. Antibiotic choice appropriateness rate was not modified by the educational intervention being very high already in phase I (91%). However, analyzing only H-CAP, we found a significant increase in antibiotic choice appropriateness rate after the educational intervention and the adherence to guidelines reached 100%. Particularly, macrolide prescriptions were prescribed inappropriately in 13.46% and this rate was halved after educational intervention.

Regarding the duration of use, educational intervention only slightly increase adherence to guidelines from 60.59% to 64.71%. However, in notH-CAP, a 36% increase in adherence to guidelines was observed in phase II. Inappropriate duration was seen only for macrolides that were used shorter than recommended.

The dose of antibiotics was concordant with the guidelines in 81.20% of the 400 evaluated doses. In the discordant doses, the dose per kilogram was lower than that recommended in 67.57% of the cases. Concordant doses were found more frequently in H-CAP than in notH-CAP being 88.01% and 67.42%. Educational intervention did not modify this rate.

In our study, no ADR was reported. The lack of compliance in reporting ADRs is one of the main weakness of the paediatric pharmacovigilance system [40]. Educational training on pharmacovigilance at the territorial level should be encouraged and may facilitate a better compliance and thus optimisation of ADRs reporting.

Even if involving a single Italian region (Liguria, Northern Italy), we regard the external validity of our findings to be high, since our data represent unselected and consecutive patients. However, due to a high heterogeneity of drug use among Italian regions [32] our findings may not reflect paediatric antibiotic prescribing in Italy as a whole and the appropriateness should be investigated in all Italian regions as part of drug utilization monitoring plan. As reported by Cartabia et al. [32], the geographic distribution is not explained by the health status of the children but by the tendency of the pediatrician to prescribe drugs. In addition, we collected data to assess pneumonia severity or other factors that may have influenced decisions regarding diagnostic testing and antibiotic management. Of note that in the first phase of this study, pediatricians were kept unaware of the present evaluation, so their prescribing of antibiotic practices could not have been influenced by the study: therefore our approach permitted objective evaluation of physicians’ prescribing habits in everyday practice. Another strength of the present paper is that the study period spanned over one-year to avoid seasonal variation in illness that may affect antibiotic prescribing.

Among limitations, data on microbiological investigation were not available in all cases, therefore some patients diagnosed with pneumonia may have had viral etiologies. However, even with the understanding that some pneumonias in our study population were likely viral, the misdiagnosis of viral pneumonias as bacterial should not have affected antibiotic selection once the physician’s intent was to treat bacterial pneumonia.

In the surveillance part of the MAREA study, a needed sample size of 840 patients (420 patients each phase) was calculated assuming an expected frequency of hospitalization of about 10% in pediatric population with a diagnosis of pneumonia, AOM and/or pharyngotonsillitis. We enrolled only 620 patients but this only slightly decreased the statistical power (from 80% to 75%) because the observed frequency of hospitalization for the three indications was higher than expected (25% vs. 10%, data not shown). Alternatively, to determine the hospitalization percentage more accurately, we should have conducted a pilot study. Being the appropriateness study strictly depend on the surveillance study (i.e. the number antibiotics prescriptions are related to the number of patients enrolled), the same sample size was kept also for the appropriateness study.

## Conclusion

Our study shows that prescribing antibiotic practices were globally sufficiently appropriate, but certain aspects deserve closer investigation, above all the widespread preference for multidrug empirical regimens or macrolide in monotherapy which in some cases seemed difficult to justify.

In addition, findings from this study underline that priority actions for improving the rational use of antibacterials in children should concentrate on education activities such as active participation of the pediatricians to training courses or availability of electronic decision support systems that may facilitate guideline-concordant antibiotic prescription.

## Abbreviations

ADR: Adverse drug reactions
AOM: Acute otitis media
e-CRF: Electronic case report form
EI: Enzyme inhibitor
H-CAP: Hospitalized pneumonia
NotH-CAP: Not-hospitalized pneumonia
SD: Standard deviation
